# Changing to an Opt Out System for Organ Donation—Reflections From England and Netherlands

**DOI:** 10.3389/ti.2022.10466

**Published:** 2022-07-04

**Authors:** N. E. Jansen, C. Williment, B. J. J. M. Haase-Kromwijk, D. Gardiner

**Affiliations:** ^1^ Dutch Transplant Foundation, Leiden, Netherlands; ^2^ National Health Service Blood and Transplant, Bristol, United Kingdom

**Keywords:** organ donation, Opt Out, Opt In, Donor Register, consent rate, consent system

## Abstract

Recently England and Netherlands have changed their consent system from Opt In to Opt Out. The reflections shared in this paper give insight and may be helpful for other nation considering likewise. Strong support in England for the change in legislation led to Opt Out being introduced without requiring a vote in parliament in 2019. In Netherlands the bill passed by the smallest possible majority in 2018. Both countries implemented a public campaign to raise awareness. In England registration on the Donor Register is voluntary. Registration was required in Netherlands for all residents 18 years and older. For those not already on the register, letters were sent by the Dutch Government to ask individuals to register. If people did not respond they would be legally registered as having “no objection.” After implementation of Opt Out in England 42.3% is registered Opt In, 3.6% Opt Out, and 54.1% has no registration. In contrast in Netherlands the whole population is registered with 45% Opt In, 31% Opt Out and 24% “No Objection.” It is too soon to draw conclusions about the impact on the consent rate and number of resulting organ donors. However, the first signs are positive.

## Graphical Abstract

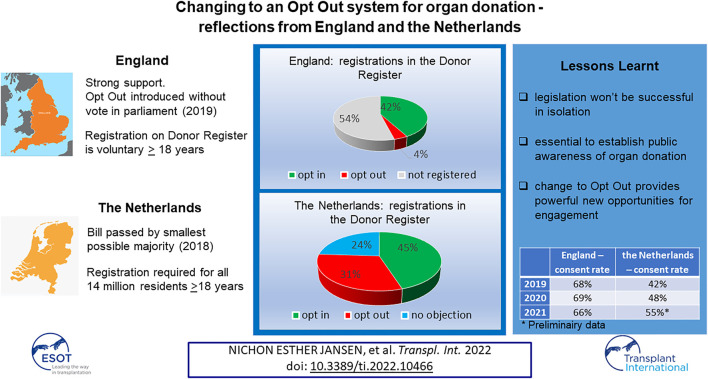

## Introduction

Recently England and Netherlands changed their consent system for deceased organ and tissue donation from Opt In to Opt Out. The aim of this article is to give insight into the process of changing the law, implementation and initial impact.

## The Donation Landscape Prior to Opt Out

### England

The modern era of organ donation in the United Kingdom (UK) commenced in 2008 with the implementation of recommendations from the Organ Donation Taskforce.^1^ Key initiatives included the creation of National Health Service Blood and Transplant (NHSBT) as a single donation organisation for the UK, resolving ethical and legal barriers to donation, and the introduction of champion roles in hospitals for donation such as the clinical lead for organ donation (normally an intensive care doctor) and a lay chair of an organ donation committee; both roles to be supported by the embedding of specialist nurses for organ donation into intensive care units. This change led to a 50% increase in deceased donations by 2013 but consent rates remained stubbornly static (1). A second national strategy in 2013 “Taking Organ Transplant to 2020” called for a revolution in consent (2). This would be achieved through marketing and media campaigns, specialist nurse training and specialisation in the family approach for consent and emphasising that the family discussion about organ donation should be a collaborative and combined effort between hospital staff and the specialist nurse for organ donation. Consent rose accordingly but not to the levels peer nations were achieving. To increase consent, societal change was required.

Wales passed Opt Out (Deemed Consent) legislation in 2013, with implementation in 2015 (3). Previously, individuals could make their donation decision known by opting in (registering) on the National Health Service Organ Donor Register (NHS ODR), or by verbally expressing to family and friends they would be willing to be a donor after death. Where no known decision in life had been made, the law gave the decision regarding organ donation to the family. Following the introduction of Opt Out in Wales, the NHS ODR was changed to allow individuals to register an Opt Out decision. Since the NHS ODR is applicable across the UK, this allowed anyone in the UK to register a decision not to donate. Applicable only in Wales, if no organ donation decision was known, the individual would be considered to have no objection to becoming a donor.

Support in England for organ donation led the government to seek to amend the consent legislation to Opt Out in 2019.

### Netherlands

In 1997 the Organ Donation Act was passed, based on an Opt In consent system for organ and tissue donation. All people aged 18 years and older received a letter from the government, asking them to register their donation preferences. The preferences allowed in the Donor Register were; “Yes, I want to be a donor,” “No, I do not want to be a donor,” “Decision by next of kin,” or “Decision by a specific person.” Ten years after implementation of the law 5.2 million (40%) of the 13 million Dutch residents, from 18 years and older, had registered their donation preferences. Despite this, there were still not enough donors to meet the number of patients on the waiting list.

In 2007, a TV show revealed in a dramatic way the need for more organ donations. In a live national broadcast, “Dutch Donor Show,” a terminal ill woman was asked to choose between three candidates and donate her kidney to that person. At the moment she announced her chosen recipient, the presenter intervened, explaining that this offer was not for real. It was a fake scenario. The potential donor was an actress but the kidney recipients on the show were genuine and on the waiting list, all fully aware of the nature of the show. Not even the Dutch Transplant Foundation or the government had been made aware of the truth. The aim of the broadcast was a wakeup call for politicians and Dutch society to do something about the shortage of organ donors for patients on the waiting list for a transplant.

Following the show, a coordination group “Organ Donation” was formed in 2007, consisting of several stakeholder organisations and led by an independent chairman. Within 1 year a Master Plan Organ Donation (4) was established based on 4 pillars: 1) changing the Opt In consent system into an Opt Out system; 2) facilitating organ donation in hospitals in a more efficient way; 3) education of the public to positively support organ donation; 4) taking away financial barriers for living organ donation. The overall aim was to increase the number of organ transplants by 25%: 15% by changing to Opt Out and 10% through improvements in donor hospitals and public information. In 2014 the Master Plan Organ Donation was evaluated, the increase in numbers of transplants was 11%.^2^

Three out of the four pillars were actively being addressed. For a more efficient way to facilitate organ donation, hospitals were divided into seven donation regions. Donation intensivists were introduced in larger hospitals together with a donation coordinator, to support and promote donation policy in a cluster of hospitals. National teams were introduced to facilitate organ retrieval in donor hospitals. Several campaigns were launched to educate the public in organ donation. The living donor program had achieved considerable success since 2008 (306–520 transplants in 6 years, 70%) by removing financial barriers and through the implementation of organisational and promotional activities (5). The only pillar that had not been addressed was changing to an Opt Out system.

## Parliamentary Process to Introduce Opt Out

### England

There had been many failed attempts to introduce Opt Out legislation to England over the last 30 years but was achieved on 20th May 2020. In October 2017 the Prime Minister stated her intention to shift “the balance of presumption in favour of organ donation” and “introduce an opt out system for donation.”

Fortuitously a parliamentarian from the opposition party had successfully had his name drawn from a legislation ballot (a system which allows a few “Private Members Bills” to be considered by parliament from a randomly chosen subset of legislation suggestions), for a new Opt Out Bill. This led to an unusual alignment of opposing political parties, working together on a new policy. Due to this cross party support, the Bill progressed through Parliament and never had to be put to a vote.

England’s Opt Out legislation built on the positive experience in Wales and Parliament was further reassured by the response to a public consultation on the draft Bill, which asked how Opt Out should be introduced. The Government usually expects between 200 and 500 responses; over 17,000 responses were received. The responses were supportive and gave a strong steer for the issues needing to be addressed.

The main issues raised by the public were: the need for autonomy and individual choice; the role of the family; the need to respect faith and beliefs through the donation process. The government worked closely with NHSBT to identify ways to ensure that these issues were addressed. Ministerial commitments also secured additional resources such as increased recurrent funding.

The final inspiration came from two young people—Max Johnson and Keira Ball. When the Bill was introduced, Max Johnson, a 9 year old boy, was in desperate need of a heart transplant. The UK media—particularly the Mirror newspaper—campaigned for the introduction of Opt Out legislation. Max’s life was saved through the gift of donation by Keira Ball, also aged nine, who tragically lost her life in a road traffic collision. The Opt Out legislation is known as Max and Keira’s Law, in their honour.

### Netherlands

On the 1st of July 2020 the Opt Out system for organ donation was implemented in Netherlands. Changing the organ donation law from an Opt In consent system into an Opt Out system had not been easy. It took more than 12 years of political discussion to reach the milestone of a majority.

In 2012 a member of the House of Representatives prepared a Bill to change the consent system into an “Active Donor Registration.” On the 16th of September 2016 the Bill was passed by the smallest possible majority in the House of Representatives, 75 members voted in favour of the Bill and 74 members against. On the 16th of February 2018 the vote in the Senate again ended in a close call, 38 senators voted in favour of the Bill and 36 members against. The Bill could only pass after a required amendment to develop a “Quality Standard Donation,” which describes the role of the doctor and the family in the donation conversation, based on the different outcomes of the Donor Register.

The Active Donor Registration means that Dutch residents without a registration in the Donor Register, 7 million, will be asked by letter to register their donation preferences (same options as in the Opt In system). If they do not respond to a first and second letter, they will receive a third and final letter with the confirmation that they will be registered as having “No Objection” to organ and tissue donation. Under the new legislation “No Objection” would legally be considered the same as a registration of “Yes, I want to be an organ donor.” Registrations can be changed 24 h a day *via* the Internet. It could therefore be argued that while the change in law was to introduce Opt Out, it has similarities to a model of mandated choice for organ and tissue donation (6).

## Implementation

### England

**FIGURE 1:**
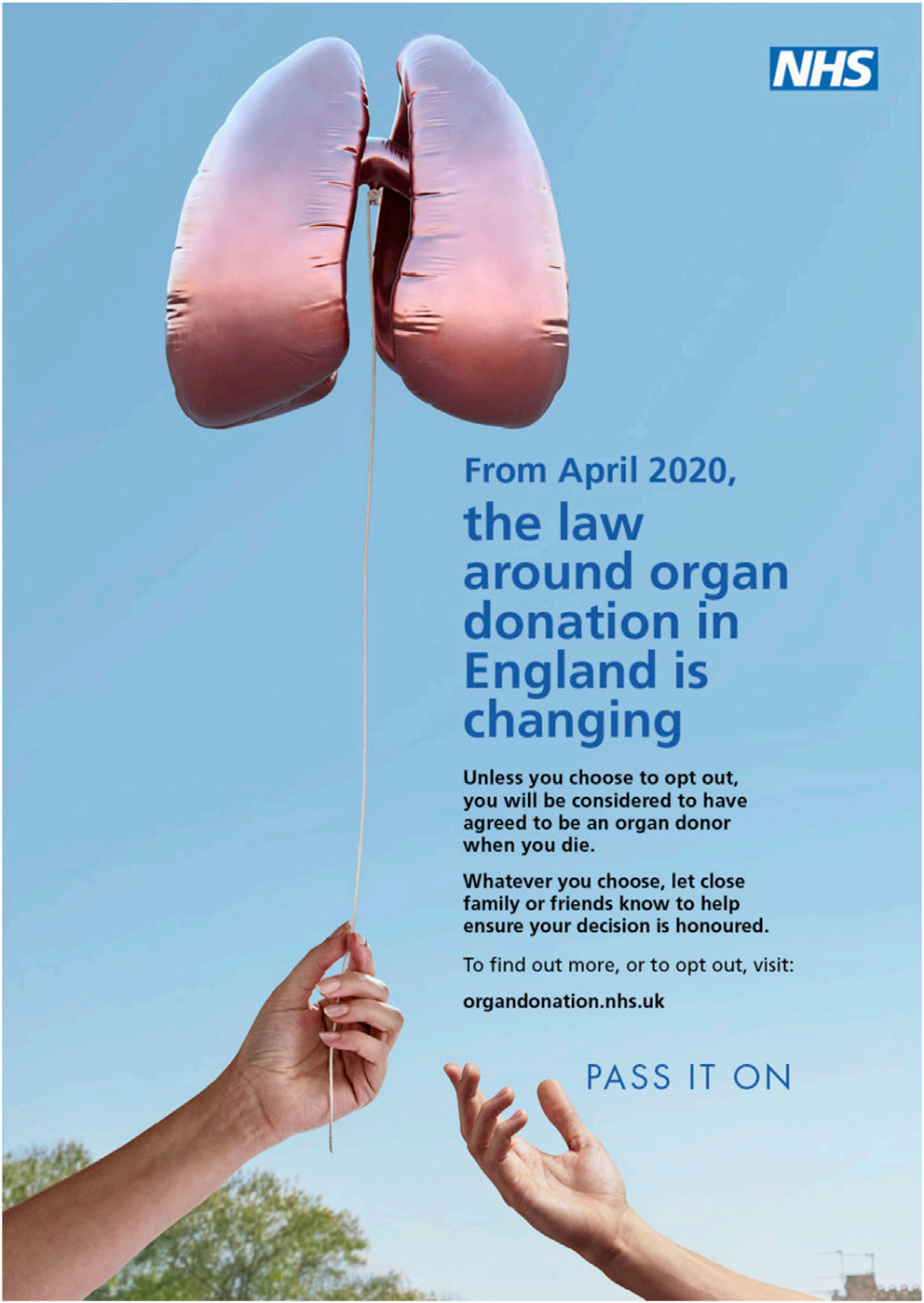
Marketing NHS new law organ donation.

The learning from Wales made it clear that there needed to be at least a year of marketing activity, so the public understood the change in legislation and what action they should take. The “Pass It On” campaign was developed to include advertising on TV, radio and social media, as well as posters and billboards. The marketing gave clear messaging as shown in Figure 1.

In the first 9 months, the advertising was kept low-level, but this ramped up significantly in the final 3 months prior to the go-live date. The onset of the COVID-19 Pandemic required the removal of the “Pass it on” slogan, but the general message remained consistent.

There was also engagement with different communities, to raise awareness of the change in law and dispel myths. Significant concern was expressed by some regarding the role of the family, the loss of autonomy and the impact on adherence to faith/belief requirements following death. Following close working with community and faith groups to discuss their concerns and identify approaches to provide reassurance, the NHS ODR was amended to enable people to record that they wanted their faith/beliefs to be taken into consideration.^3^ Community champions were provided with materials to raise awareness including a guide to the journey through intensive care and organ donation.

Work was also underway to ensure the clinical donation community were aware of the change in law and its potential impact. Codes of practice were developed by the Human Tissue Authority, to interpret the legislation and provide best practice guidance (7).

**FIGURE 2:**
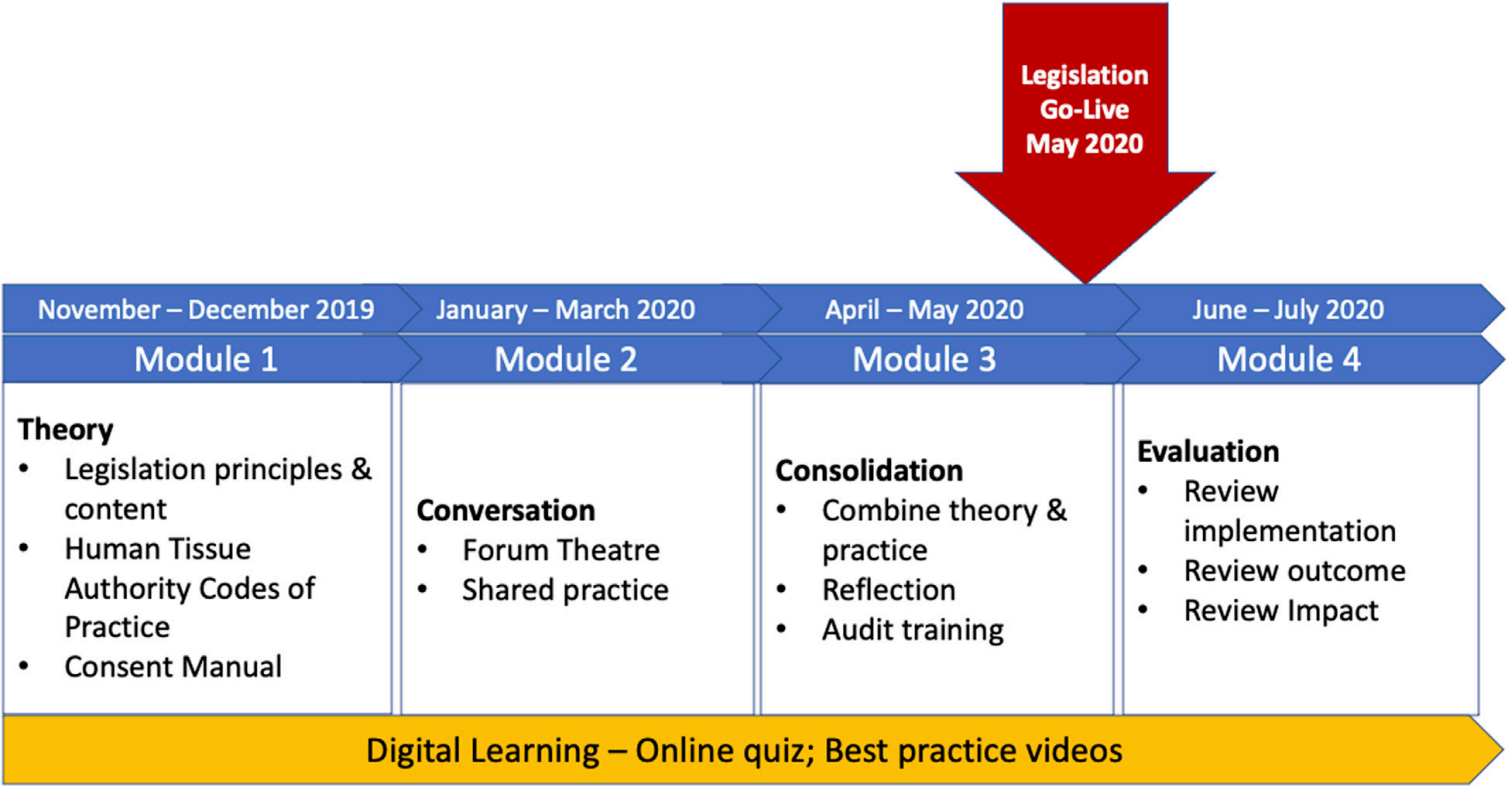
Training programmes new donor law for Specialist Nurses for Organ Donation.

In the UK the main healthcare professional who makes the family approach to discuss organ donation is the Specialist Nurse for Organ Donation. The government provided funding for recruiting 27 additional nurses and training programmes were established for all Specialist Nurses. This included four modules, as shown in Figure 2. As the pandemic evolved these moved to virtual training.

The digital infrastructure was also changed to support the legislation. The NHS ODR already included the ability to opt out, as well as opt in, as a result of the Welsh legislation in 2015. The NHS ODR became integrated with the new NHS app, meaning that for the first time people could see and directly amend their own record. Online consent forms and associated paperwork were amended to enable records and databases to capture where a deemed consent scenario applied and the outcome.

The cost of implementation to NHS Blood and Transplant was £7.8 million for operational activity including funding the programme, changes to digital infrastructure and new staff appointments and training. Marketing allocation was £11.7 million with £7 million being spent in the last year before implementation. This was England’s largest single organ donation marketing budget. Other costs to develop and implement the legislation change incurred, e.g., government, local organ donation committees.

### Netherlands

The law “Active Donor Registration,” Opt Out system, was implemented on the 1st of July 2020, which was during the COVID-19 pandemic, just after the first wave. Due to this pandemic the Minister of Health, Welfare and Sports (VWS) decided to postpone the implementation process, including media campaigns, until the 1st of September 2020. The process of sending letters to 7 million residents without a registration, lasted until the end of July 2021. At that date the donation preferences of the whole population from the age of 18 years onwards, 14 million in total, were registered in the Donor Register.

**FIGURE 3:**
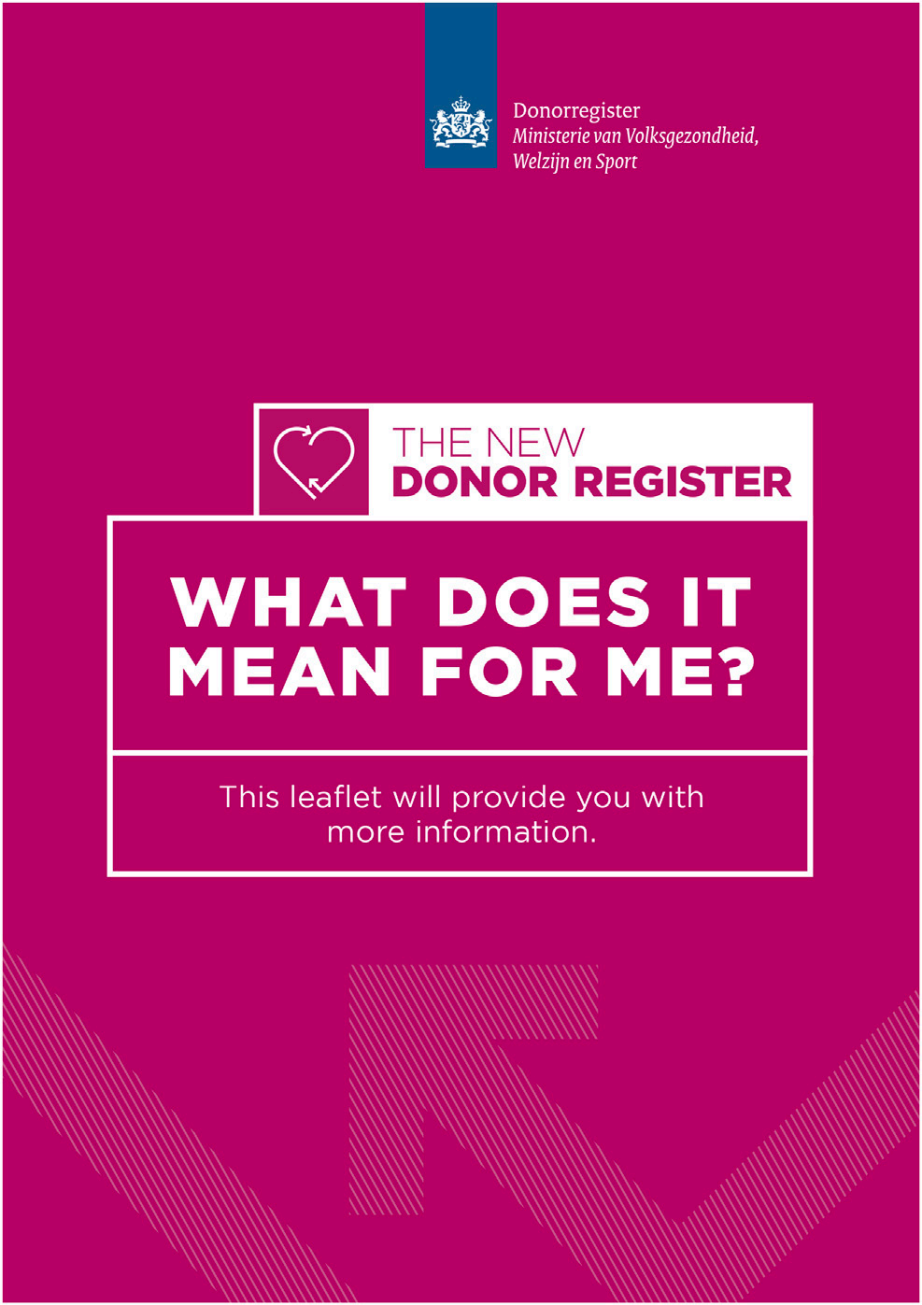
Front cover of a leaflet sent by the Ministry of VWS explaining the new organ donation law.

To prepare the Dutch population for the change of law the Ministry of Health, Welfare and Sports (VWS) was responsible for the public campaigns (e.g., see Figure 3) and the Dutch Transplant Foundation for educating medical professionals.

The Ministry of VWS released a significant amount of money to implement the new law. In total nearly €40 million; €24 million for sending the letters using the existing structure of the tax authority and €15 million for media campaigns. It was a major communication challenge, as the law impacted on everyone in Netherlands. The aim was to achieve a minimal level of knowledge about the new law for all different types of residents, for example; people with mentally impairment, people with low literacy, inmates, people with a migration background, homeless people, elderly people in nursing homes, blind and visually impaired, deaf and hearing impaired.

The mass media campaigns were divided into two phases. The first phase started in 2019 and aimed to inform the public about the new donation law and motivate and activate Dutch residents, without a registration, to actively register their donation preferences. In this phase registration was voluntary. The second phase started in 2020 by sending people letters to register their donation preferences. If they did not respond they would be legally registered as having “No Objection” to donation, which could be changed at any time. Since registration in this phase was required, the information in the campaign was explicitly neutral, not giving a direction to any resident. In addition to the mass media campaigns there were initiatives to reach out to target groups, for example faith groups, elderly in nursing homes, illiterate people, etc. (see Table 1). Meetings were organised in a small scale setting to get in close contact with people who may not be reached by mass media.

**TABLE 1:** Summary of implementation measures in England and Netherlands.

	England	Netherlands
Media/Marketing campaigns	TV commercials	TV commercials
	Radio commercials	Radio commercials
	Online and social media campaigns	Online and social media campaigns
	Billboards	BillboardsDoor-to-door newspapers/flyers
Targeted actions	**Before the law**	People with low literacy: www.hoewerktorgaandonatie.nl
	Focus groups informed implementation e.g., Paediatric, Socio-economic, Faith and beliefs	People with learning difficulties: www.vgn.nl/nieuws/update-16-3-faq-nieuwe-donorwet-voor-zorgorganisaties
	**During implementation**	People with a migration background: www.donorregister.nl/voorlichtingsmateriaal/arabic
	Faith and beliefs engagement	Deaf and hearing impaired: www.donorregister.nl/voorlichtingsmateriaal/nederlandse-gebarentaal
	Community engagement	Homeless people:
	Ethicists e.g., British Medical Association ethics committee, Nuffield Council of Bioethics	Information packages were sent to social care counselors and social relief institutions
	Organ Donation Regional Collaboratives	People in nursing homes:
	Intensive Care Society State of the Art 2019 drama production “Choose your own organ donation approach”	Tailor-made information campaign aimed at intermediaries (informal carers, carers, family)
		Blind and visually impaired people:
		Audio file was distributed to interest groups of blind people
		Inmates:
		Information packages were distributed to prisons
		Information meetings in the 4 largest cities with community leaders of diverse populations—Donor dialogue teams: www.inclusia.nl/projecten-2/50-jaar-migratie/
School education	Published teaching materials and organ donation became a mandatory part of secondary school curriculum	Guest lectures
		Online education package www.donorwise.nl
Call centre	Call centre handled calls from the public regarding change in the law and requests to record a decision on the NHS Organ Donor Register	Call centre handled calls from the public regarding change in the law and registration in the National Organ Donor Register
	20,000 calls between May 2019–December 2020 to a dedicated line	46,000 calls between July 2020 until May 2022
	In addition to 46,000 calls received via our standard NHS Organ Donor Line (on any topic)	In addition 40,000 calls received on any topic about donation
Training medical professionals	Face to Face (pre-COVID), then virtual online training	Online training
	Video examples can be seen at: https://www.odt.nhs.uk/deceased-donation/best-practice-guidance/consent-and-authorisation/	Practical training in the hospitals www.transplantatiestichting.nl/medisch-professionals/donatiegesprek

The Dutch Transplant Foundation was responsible for preparing medical professionals for the new donation law, including how to request for donation in accordance with the Quality Standard Donation. Training programmes were developed, not only for intensivists who approach families for organ donation, but also for physicians who are involved in tissue donation. Furthermore, the website of the Dutch Transplant foundation provides an interactive decision tree for the correct steps to approach families based on the donor registration. There is also a “Frequently Asked Questions” section on the website to help doctors.

## Impact on Engagement

### England

Whilst the change in law was considered a positive move to increase organ donation, it is the conversation, debate and education it prompted that will lead to the biggest benefits. The campaign encouraged people to consider organ donation, register a decision and speak to their family. The data would suggest that people followed this approach, as the numbers on the NHS ODR continue to rise and after over 17 months since the original campaign, public awareness of the law change is sustained at around 70%.

Further materials are also demonstrating the initial discussions held with stakeholders are having a longer term impact and supporting peer education. For example, in September 2021 the Office of the Chief Rabbi launched new education materials to raise awareness of organ donation and the impact of the change in law.^4^ Education is also being taken forward in schools, with the law prompting the introduction of blood, organ and tissue donation into the mandatory curriculum for secondary school children. Patient support groups and donor families had been lobbying for this change for nearly 2 decades without previous success. NHSBT supported this by providing teaching resources.^5^

### Netherlands

It was important that the public were aware of the impact of the new law and that they were required to be on the Donor Register. If they had not responded to the letters they would be registered as having “No Objection.” In practice, although consent for donation is given by the donor, families need to know each other’s donation preferences as donation will only take place after informing the next of kin. All media campaigns launched by the Ministry of VWS about the Opt Out system were aimed at encouraging people to talk about organ and tissue donation and register their preferences. The effects of the campaigns were monitored, before and during the implementation of the Active Donor Registration. The outcome of all campaigns is that 85% of the population has knowledge about the new law.^6^

To tackle the challenge of informing all residents in Netherlands, several “targeted” actions started. Special Donor Dialogue teams were trained, to raise awareness of the new law with community leaders of diverse populations. Unfamiliarity with the subject of organ and tissue donation and language barriers, such as low literacy, can play a role in the number of registrations in the Donor Register.^7^ The Donor Dialogue team organised meetings in several neighbourhoods in the four largest cities in Netherlands, to discuss donation and how this relates to the culture and religion of the participants.

For many years, with funding from the Ministry of VWS, the Dutch Transplant Foundation has run “Donorwise,” an education package for primary and secondary schools, and a yearly campaign to encourage those turning 18 to register their donation preferences. Following the law change the yearly campaign now “requires” those turning 18 to register a donation decision.

## Impact on Number of Registrations in the Donor Register Before and After the Change of Law

### England

**FIGURE 4:**
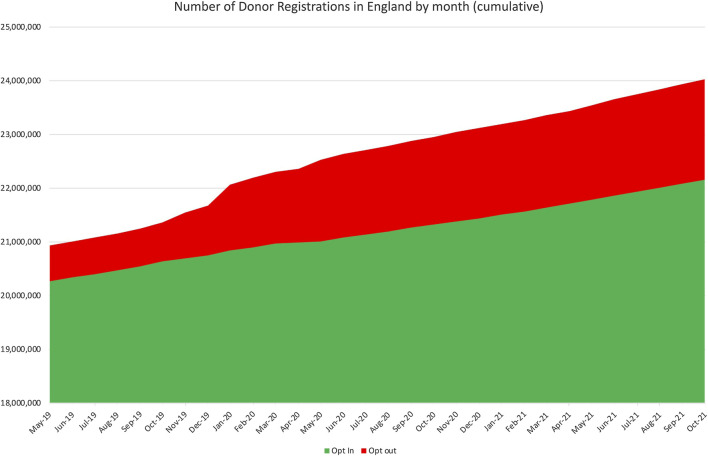
Number of Donor Registrations in England by month; May 2019–October 2021 NB. It is legally possible to appoint a representative to make a decision on your behalf, this requires in the UK a written application, and only 139 people (roughly equivalent to the yellow in Figure 5) from England have done so. Population of England aged 18 or over: 52,383,965.

Since Wales implemented Opt Out in 2015, anyone in the UK has been able to register an opt-out on the NHS ODR. As of 20 September 2021, 3.3% of the English population (3.6% of the population 18 years or over) had registered an opt-out decision on the NHS ODR, compared to 6.2% in Wales (Opt-in: England: 39.2%; Wales 42.7%). The largest spike in opt-out registrations occurred in January 2021, 5 months before the law was implemented, when 295,000 individuals registered an opt-out decision. This was associated with fake news circulating on social media. The next highest month for opt-out registration was 144,000 corresponding to May 2021, when the law was implemented. In the 5 months since the law was implemented there have been no peaks in opt-out registrations and an average of 23,000 people register an opt-out each month compared to 73,000 opt-in (Figure 4).

### Netherlands

**FIGURE 5:**
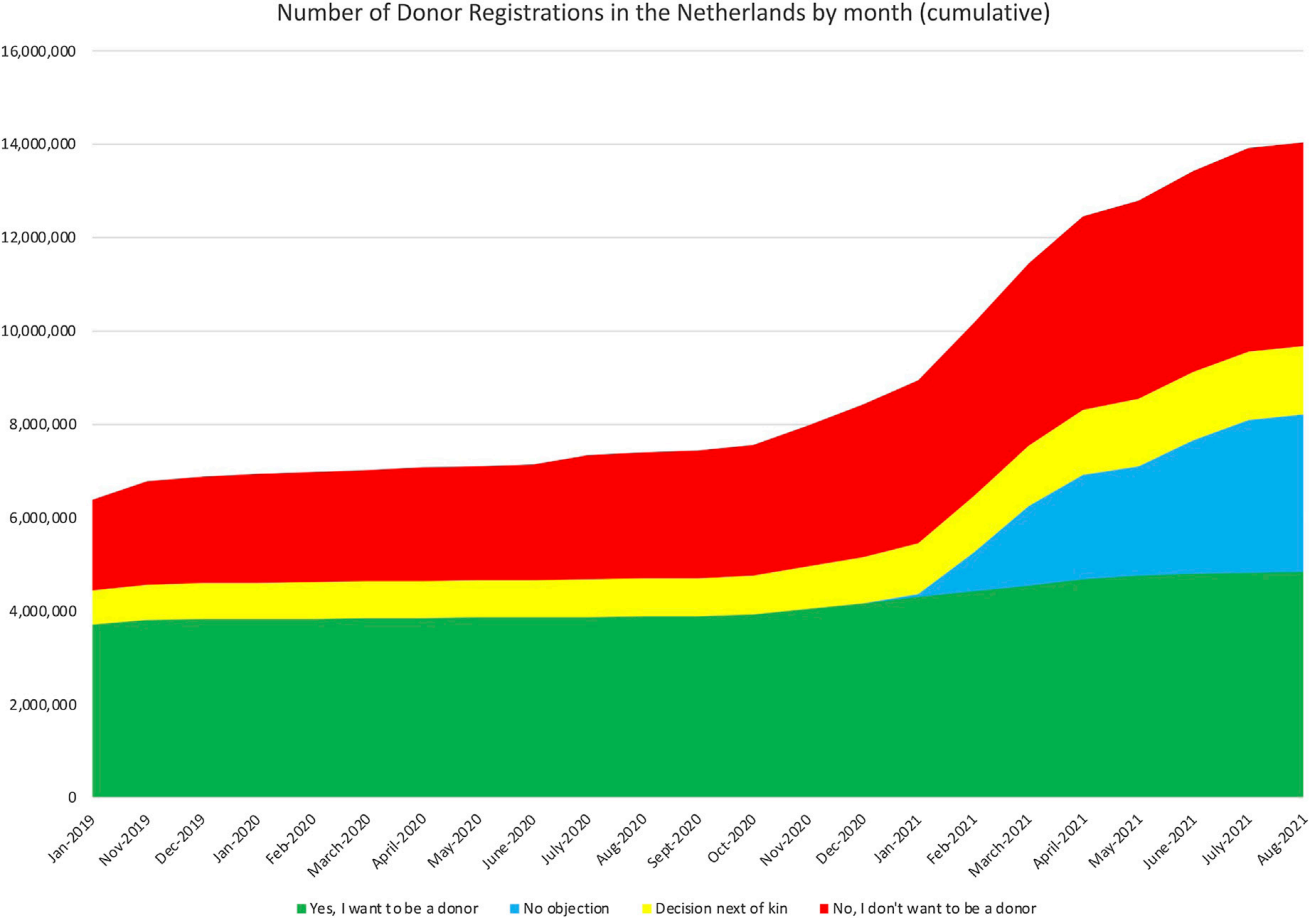
Number of Donor Registrations in Netherlands by month; January 2020–Augustus 2021.

When the process of sending registration letters to 7 million non-registered residents was completed, by the end of July 2021, the impact on the number of registrations compared to the beginning of 2020 was as follows.^8^ The registration “Yes, I want to be a donor” increased by nearly one million (from 3.8 million to 4,8 million), the number of registration “No, I don’t want to be a donor” increase even more (from 2.3 million to 4.3 million), the “Decision by next of kin”/“Decision by a specific person” showed an increase from 0.8 million to 1.5 million. The number of people who did not respond to the letters, asking to register their donation preferences, was 3.3 million. They are registered with “No Objection” to donation. Overall, the number of active registrations increased by 3.7 million, from 6.9 million in January 2020 to 10.6 million in August 2021. This means that 75% of the population registered their donation preferences. Adding the 3.3 million “No Objection” registrations means that all 14 million people from 18 years of age and above are registered in the Donor Register (Figure 5). This demonstrates that the communication was effective and that people were considering donation and recording their decision. This achieved a key aim of the new donor law to know the donor preferences of the whole population of 18 years of age onwards. This record provides clarity to the potential donor family when approached for organ donation.

An overall comparison of the Donation Consent System between England and Netherlands is shown in Table 2.

**TABLE 2:** Comparison of England and Netherlands—donation consent system.

	England	Netherlands
Former consent system	Opt In. Consent from family if no Opt In registration	Opt In. Consent from family if no Opt In registration
Parliamentary process	Private Members Bill with no formal objections raised as it progressed through Parliament. No vote required	The Bill was passed with the smallest possible majority in the House of Representatives (1 person) and Senate (2 people)
Aim of changing the consent system	To better reflect the public support for organ donation and increase the consent rate	To know the donation preferences of the Dutch population. And for families to know the preferences of the donor when approached for donation
Messaging from the Government	Positive messaging highlighting: the law is changing; a call to action to make your organ donation decision and inform your family; promoting the benefits of organ donation for patients and donors	Neutral messaging highlighting: register your preference and inform your family; implications explained if individuals, who were not registered already, did not respond to the mailout letters
Registrations in the Donor Register	Voluntary registration	Required registration
	Of the Population of England aged 18 or over	Of the Population of Netherlands aged 18 or over
	42% opt in, 4% opt out, 54% not registered,	45% opt in, 31% opt out, N/A not registered,
	N/A no objection	24% no objection
Consent in practice	Families must be consulted	Families must be consulted
	Donation not enforced in the face of family objection	Family arguments opposing donation respected

N/A, not applicable.

## Impact on Consent Rates and Donor Numbers

### England

The experience from the introduction of Opt Out in Wales in 2015 was that change to consent rates did not happen immediately (8). However, in a study comparing Wales to England, after 3 years (2015–2018) the chance of consent in Wales was double that seen in England and donor numbers had risen more rapidly (Wales: 18.0 to 28.9 donors pmp; England: 20.0 to 24.3 donors pmp) (9). Interestingly, compared to Donation after Brain Death, the change in consent rates for Donation after Circulatory Death did not reach statistical significance.

By September 2020 England had its highest consent rate on record (70.3%) but since then there has been a steady decline in consent, see Table 3. Judging any change is extremely difficult owing to the COVID-19 pandemic and its impact on intensive care and society (10). Similarly, although donor numbers have risen in England this most likely reflects donation numbers recovering from the large drop in 2020 caused by the pandemic (11).

**TABLE 3:** Comparison of England and Netherlands—annual consent rate.

Calendar Year	England		Netherlands	
	Implemented 20th May 2020		Implemented 1st July 2020	
	Annual consent rate (%)	Monthly range (min %, max %)	Annual consent rate (%)	Monthly range (min %, max %)
2019	68	(64, 74)	42	Not available
2020	69	(65, 74)	48	Not available
2021	66	(58, 72)	55^a^	(43, 63)

a Preliminary data.

### Netherlands

The first registrations of “No Objection” to donation only commenced in January 2021. It is too soon to draw definitive conclusions about the impact of the new donation law on the consent rate and organ donor numbers. Although we see positive signs, see Table 3.

Also the impact of COVID-19, especially the first wave, had a dramatic effect on the number of organ donors and number of transplantation. The total amount of all organ transplants decreased with 67% (12).

More time is needed to adjust to the new donation law, not only for the public but also for the doctors. The effect of the new law will be monitored closely in the coming years. Like the experience in England, unpicking the legislation change with the impact of the COVID-19 pandemic is extremely challenging.

Another benefit observed in Netherlands following the new donation law was the effect on the number of tissue donors. Tissue donors increased by 26% (from 1923 tissue donors in 2020–2427 in 2021) with consent rising from 20% in 2020 to 43% in 2021. What we saw in the former years is that “no registration” in the Donor Register was a very difficult situation for the donor family to respond to. We see now that consent registration based on a registered “no objection” gives a positive direction for the donor family, knowing the preferences of their loved one to donate. This results in a higher consent for tissue donation.

## Lessons and Recommendations

From our experiences in England and Netherlands we would share the following lessons from introducing Opt Out:

(1) Legislation won’t be successful in isolation - before the law changes it is essential to have an effective operational infrastructure for organ donation and an established public awareness of organ donation.
(2) Acceptance of the law will be easier if there is already widespread political and societal support for introducing Opt Out.
(3) Implementation of a required registration in the Donor Register for the whole population of 18 years onwards, as in Netherlands, poses different challenges in disseminating the message/campaigns.
(4) Implementing legislative change into practice requires a comprehensive plan covering: training of healthcare professionals, codes of clinical practice, digital infrastructure (e.g., Organ Donor Register changes), public awareness campaigns and engagement with stakeholders from all areas of society.
(5) The legislation can act as an enabler for wider change and engagement. For example by increasing donation funding for staff and public campaigns, changing school curriculum to make donation education mandatory, greater involvement of faith and community groups.
(6) There is a responsibility to monitor and evaluate the impact of Opt Out and share findings with the world wide donation community.

## Conclusion

It’s still too early to tell what the final impact of the introduction of Opt Out into England and Netherlands will be. We hope the reflections shared in this paper give insight into changing the consent system and help for any other nation considering likewise.

## Data Availability

The raw data supporting the conclusion of this article will be made available by the authors, without undue reservation.

## Footnotes

1
https://nhsbtdbe.blob.core.windows.net/umbraco-assets-corp/4245/organsfortransplantstheorgandonortaskforce1streport.pdf.

2
https://zoek.officielebekendmakingen.nl/kst-28140-85.html.

3
https://www.nhsbt.nhs.uk/news/faith-and-beliefs-declaration/.

4
https://chiefrabbi.org/all-media/changes-to-english-law-on-organ-donation-faqs/.

5
https://www.nhsbt.nhs.uk/how-you-can-help/get-involved/download-digital-materials/donation-teaching-resources/.

6
https://www.rijksoverheid.nl/documenten/kamerstukken/2021/07/07/kamerbrief-over-afronding-implementatie-nieuwe-donorwet.

7
https://inclusia.nl/projecten-2/50-jaar-migratie/.

8
https://www.cbs.nl/nl-nl/nieuws/2021/40/3-7-miljoen-meer-actieve-registraties-in-donorregister-sinds-2020.
